# Ethnic inequalities in time to diagnosis of cancer: a systematic review

**DOI:** 10.1186/1471-2296-14-197

**Published:** 2013-12-23

**Authors:** Tanimola Martins, William Hamilton, Obioha C Ukoumunne

**Affiliations:** 1 University of Exeter Medical School, Veysey Building, Salmon Pool Lane, Exeter, UK

**Keywords:** Health inequality, Ethnicity, Cancer, Diagnostic delay, Diagnostic intervals

## Abstract

**Background:**

Minimising diagnostic delays in cancer may help improve survival. Ethnic minorities have worse outcomes in some cancer types when compared to the majority; this may relate in part to differences during the diagnostic phase. Only a few British studies have specifically explored this relationship, and no synthesis of these exists. The present study aimed to systematically review evidence on ethnic inequalities in cancer diagnosis, focussing on patient and primary care intervals of diagnosis.

**Methods:**

Six electronic databases were searched. Included studies were those conducted in the UK or elsewhere (where access to healthcare is comparable to the NHS) and those that described a time element during diagnosis. Study quality was evaluated using the Critical Appraisal Skills Programme (CASP) checklist for cohort studies and synthesis method was narrative.

**Results:**

Seven of 8,520 studies retrieved by our search met the review criteria; six conducted in the UK, and one in New Zealand. Five (including one covering several sites) focused on breast cancer, one on prostate, and one on oesophagogastric cancer. The studies employed different methods of ascertainment and definition of ethnic groups and defined diagnostic delay in a non-standardised way; therefore, narrative synthesis was performed. In breast cancer, three studies reported longer diagnostic intervals among ethnic minorities and two found no evidence of differences by ethnicity. There was some evidence of longer diagnostic and referral intervals among ethnic minorities in oesophagogastric and colorectal cancers, but no evidence of this in prostate, non-Hodgkin’s lymphoma, lung, and ovarian cancers. None of the studies identified shorter patient or primary care intervals in ethnic minorities.

**Conclusions:**

Existing studies provide insufficient evidence to confirm or refute ethnic inequalities in diagnostic intervals of cancer. Further studies are necessary to examine common cancer types including those frequently found in ethnic minorities (in addition to those covered here) and using current definitions of intervals in cancer diagnosis.

## Background

Minimising delays in cancer diagnosis - particularly, patient and primary care delays - may help improve cancer outcomes in the UK, which at the moment lag behind most other countries in Europe [1,2]. Recent studies estimate that 5,000 to 10,000 cancer deaths could be prevented annually in the UK if efforts to expedite diagnosis succeed [1,2]. As some ethnic minorities suffer more illness and report worse experience of the National Health Services (NHS) [3], it is possible this will extend to cancer diagnostics, contributing to the UK’s poor outcomes. Few UK studies have specifically explored this relationship, with most evidence coming from the US [4]. The findings of these studies may not be generalisable to the UK, given the differences in the organisation and operations of the US and UK health care systems in addition to the differences in nature and composition of ethnic groups.

Access to health care in the UK is universally free at the point of delivery with an ethos of equity in service provision. In theory, this should reduce any disparity in cancer diagnostics. There is, however, a greater incidence of advanced-stage diagnoses of female breast cancer and prostate cancers among ethnic minority groups compared to the majority [5,6], although these studies did not report an explanation for their findings. The reason for such disparities in disease stage is unclear given that pre-referral consultation rates are markedly higher among ethnic minority groups with cancer [7]. It is unknown whether this is the case for other cancer sites, including those found to be more common in ethnic minority groups - e.g., mouth, liver, stomach, oesophagogastric and ovarian cancers [8,9].

### Aim of review

**Figure 1:**
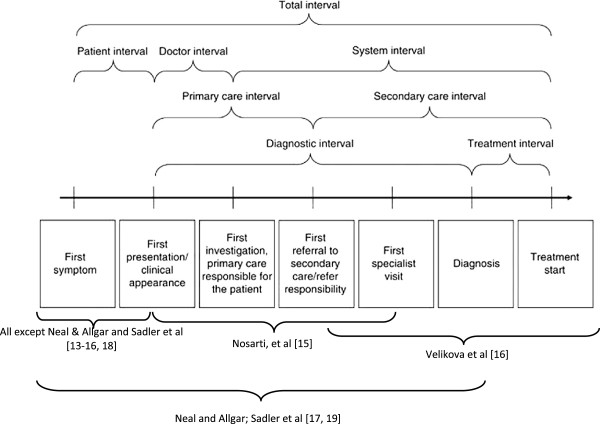
Milestone and time intervals from symptoms onset to treatment.

The present study aimed to gather and critically appraise existing evidence on inequalities in cancer diagnosis by ethnic groups in the UK and in countries with a similar health care system - in terms of costs, availability and access [10], specifically examining literature reporting time to diagnosis of the cancer. We used the schema of intervals in cancer diagnosis described by Olesen and colleagues, and illustrated in Figure 1[11].

## Methods

Between 23rd February and 6th March 2012, a systematic search of six electronic databases (EMBASE, MEDLINE, PsycINFO, Web of Knowledge, CINAHL and Campbell Collaboration) was performed. Table 1 includes the list of search terms used. Inclusion criteria were: studies focused on adult primary cancers; published from January 2000 onwards - marking the start of major reforms in cancer diagnosis in the UK; investigated ethnic differences in the interval between symptom onset and GP presentation or the interval between GP presentation and referral or diagnosis. Exclusion criteria were: studies focused on secondary cancers; cancer incidence and survival; cancer genetics; screening; prevention; treatment or co-morbidities; studies from America (other than Canada), Africa and Asia, Middle East and Eastern Europe. Our final list of countries largely matches those countries within the International Cancer Benchmarking Partnership, which seeks to explain international differences in cancer outcomes [1]. Two reviewers (TM and CS) independently reviewed the title, abstract and full text articles (where necessary) to determine studies inclusion eligibility. Studies that appeared to meet the inclusion criteria or where a decision could not be made based on the title and/or abstract were selected for full-text review to identify those for the final analysis.

**Table 1:** Review search terms

**Population**	**Exposure**	**Comparison**	**Outcome**
Terms relating to cancer : Cancer, Neoplasm, Malignant Neoplasm, tumour, Malignant tumour, Astrocytoma, Adenocarcinoma, Glioma, Mesothelioma, Medulloblastoma, Myeloma, Melanoma, Neuroblastoma, Sarcoma, Nonmelanoma, Osteosarcoma, Teratoma, Seminoma, Hodgkin, leuk?emia, Lymphoma, Retinoblastoma	Terms defining ethnic minorities in the UK: Ethnic* Race* Cultural groups, white Irish, other whites, African British, Black* Black Caribbean, British Caribbean, Asian*, Indian, British, Indian, Pakistani, British Pakistani, Bangladeshi, British, Bangladeshi, Chinese, Mixed race	Ethnic majority in the UK: Ethnic* Race* Cultural groups, White British	Terms relating to intervals of cancer diagnosis: Duration of symptom; Interval of symptoms; Time; Delay; Late; Postpone; and Wait to Symptom, Presentation, Attendance, Consultation, Appointment, Diagnosis, Detection, Treatment, Intervention, Referral. Other terms; Gate keeping, primary care

Given the methodological heterogeneity of the studies, a quantitative synthesis was not possible, so we performed a narrative synthesis, using the framework of Rodgers and colleagues [12]. Data from included studies were extracted, collated and tabulated by a single extractor (TM). The primary outcome was the interval between first symptom experience and eventual diagnosis, subdivided where possible into patient delay and primary care delay [13], with the main explanatory variable being ethnicity. The only subgroup analysis planned was by cancer site; however, the heterogeneity of the studies precluded formal meta-analysis, so we performed our narrative synthesis using the cancer sites stratified by diagnostic intervals studied.

### Quality assessment

This used the Critical Appraisal Skills Programme (CASP) checklist for cohort studies, interpreted to fit our study topic (see rows 1 and 2 in Table 2 for details). Each retrieved article was independently appraised by two reviewers (TM and OU) and was classified as either “satisfactory”, “medium” or “high” quality paper depending on the extent to which the checklist items were met, and also on the level of concerns raised about their methodology in general. For instance, a concern was raised where authors classified ethnic groups differently to the contemporary ethnic classifications in their population.

**Table 2:** Quality of studies

**CASP question**	***Was the cohort representative of a defined population?***	***Was everybody included***	***Was the exposure accurately measured to minimize bias?***	***Was the outcome precisely measured to minimize bias?***	***Have the authors identified and adjusted for all key confounding factors?***	***How precise are the results?***	***Overall quality***
Adapted question	Unchanged	*Were all eligible cancer patients studied?*	*Was ethnicity defined according to contemporary groupings?*	*Was diagnostic intervals measured to Olsen and colleagues framework?*	Unchanged. Key confounders include: age, gender, SES, co-morbidity, healthcare system, family history of cancer and tumour growth rates	Have they presented estimates of association along with the confidence intervals? Are the confidence intervals narrow?	Unchanged
Rajan et al. (2011) [14]	Met	Unmet	Partially met	Met	Unmet	Unmet	Sat
Meechan et al. (2002) [15]	Met	Partially met	Met	Met	Unmet	Unmet	Sat
Velikova et al., (2004) [16]	Met	Partially met	Unmet	Partially met	Partially met	Partially met	Sat
Nosarti et al. (2000) [17]	Met	Met	Met	Partially met	Partially met	Unmet	Sat
Neal and Allgar (2005) [18]	Met	Met	Met	Partially met	Partially met	Partially met	Med
Sadler et al. (2009) [19]	Met	Met	Met	Partially met	Unmet	Unmet	Sat
Metcalfe et al. (2008) [20]	Met	Partially met	Partially met	Met	Partially met	Met	Sat

Note: *Sat satisfactory; Med medium quality paper.*

## Results

### Study characteristics

**Figure 2:**
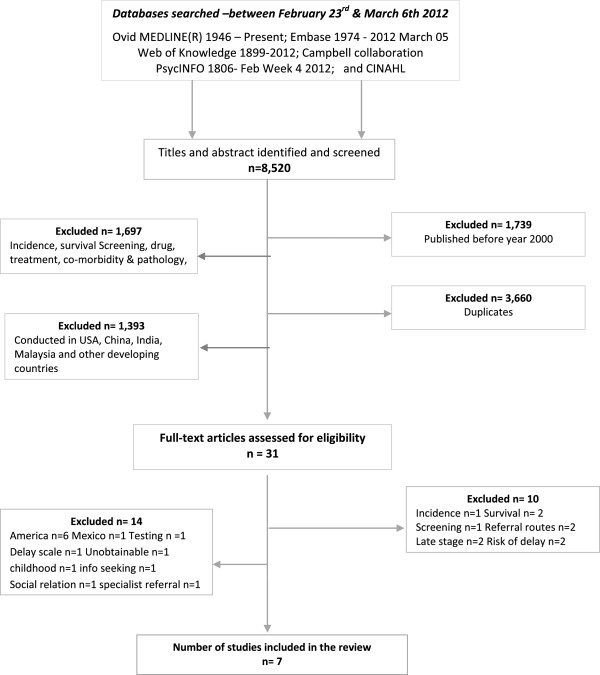
Flow chart of study selection process.

In total, 8,520 articles were identified from the search strategy. After screening the titles and abstracts, and removing the duplicates, 8,489 irrelevant articles were excluded. The remaining 31 articles were then retrieved for full text review, and seven met the inclusion criteria and were included in the analysis (see flow chart in Figure 2).

Characteristics of the selected studies are shown in Table 3 with results of the quality assessment in Table 2. All seven studies were observational, with retrospective cohort designs, using hospital records, cancer registry records, survey questionnaires and in-depth interviews. Six were carried out in the UK, and one in New Zealand. Five studies [14-18] investigated ethnic differences in breast cancer diagnosis while the remaining two focused on prostate [20] and oesophagogastric cancer [19] respectively. Neal and Allgar studied several other cancer sites in addition to breast: lung, prostate, colorectal non-Hodgkin’s lymphoma (NHL) and ovarian [18].

**Table 3:** Characteristics and findings of reviewed studies

***Author***	***Country***	***Title/aim***	***Site***	***Sample size (n) gender age***	***Method***	***Ethnic groups***		***Relevant outcome measures***	***Results***
						**Exposure**	**Comparison**		
Rajan et al. (2011) [14]	UK (West Yorkshire)	To improve knowledge about late presentation and management of breast cancer among South Asian women.	Breast	n = 1,630 (36 South Asian women) all female median age = 53.5 years	Retrospective: Breast cancer waiting list database and case notes	South Asian women; Indians and Pakistanis	None; throughout the paper, including the title, the authors imply South Asian women had more delay	Duration of breast symptoms prior to presentation within primary care	45% of Asian women delayed symptoms beyond 8 weeks before visiting their GP
Meechan et al. (2002) [15]	New Zealand	Delay in seeking medical care for self-detected breast symptoms in New Zealand women.	Breast	n = 85 all female mean age =38 years	Retrospective: questionnaire & patient record	Minority New Zealanders - Maori, Pacific & Asian/Indian	European New Zealanders	Patient delay	No difference in patient delay by ethnicity.
Velikova et al. (2004) [16]	UK (South Yorkshire)	To describe the effect of ethnicity on tumour stage, treatment, patient and providers delays in diagnosis of breast cancer	Breast	n = 16,879 all female mean age = 49.7 years in Asians and 62 years in non-Asians	Retrospective: Cancer registry data	South Asian	Non-Asian	Patients and providers delays to diagnosis	After adjusting for, age, SES and health care settings; patient delay was longer in Asian than in non –Asian women (median of 61 days vs. median of 31 days, P = 0.005)
Nosarti et al. (2000) [17]	UK	To identify factors associated with delay in presentation and assessment of symptomatic breast cancer	Breast	n = 692 all female median age = 49 years	Retrospective: Interview, GP & hospital records	African Afro-Caribbean and Asian	British and other white	Patient and system delay	Ethnicity were non-contributory to patient delay in breast cancer
Neal and Allgar (2005) [18]	UK	To explore the relationship between socio-demographic factors & delays in the diagnosis of six cancers	Breast, lung, colorectal, prostate NHL, and ovarian	n = 65,192 male & female all age groups	Retrospective: Analysis of the National Survey Data	Blacks - Africans, Caribbean & others blacks. South Asians -Indian, Pakistani, Bangladeshi, others.	Whites	Total, pre-hospital, referral and secondary care delay	After adjusting for marital status, gender, age and SES, Asian and black had longer pre-hospital delays for breast cancer in women (P = 0.001) and longer referral delay for colorectal cancer (P = 0.02). No evidence of difference for lung, prostate, NHL and ovarian.
Sadler et al. (2009) [19]	UK (Birmingham)	The effect of ethnicity on the presentation and management of oesophageal and gastric cancers: A UK perspective	oesophageal & gastric	n = 244 male & female median age = 71 years	Retrospective: Case-note audit	Asians and Blacks	Caucasians	Referral routes and total diagnostic interval	Asians and Blacks compared to Caucasians were less likely to be diagnosed within 3 months of symptom discovery (P = 0.03) and less likely to take the optimal route to diagnosis (p = 0.01).
Metcalfe et al. (2008) [20]	UK	To examine the pathways followed by black and white men to prostate cancer diagnosis	Prostate	n = 1,866 men median age = 67.9 years in blacks 73.3 years in whites	Retrospective: Questionnaire, hospital records and cancer registry data	Black men	White men	Delay between onset of symptoms and first GP presentation.	After adjusting for age and hospital centre, no significant difference between white and black men in patient delay (odds ratio: 0.82; 95% CI: 0.57 to 1.19)

Five studies [14-17,20] investigated ethnic differences in patient delay, that is, delay occurring in the interval between first symptom and first GP presentation (see Figure 1). Two of these [16,17] also examined ethnic differences in delay following the first GP presentation, but their definitions of these time periods differed. Nosarti et al. defined delay occurring in the interval between the first GP presentation and first specialist visit as system delay [17]. Velikova et al. defined the interval between first GP referral and the first hospital visit as provider’s delay [16]. Neither of these correspond with current definitions [13]. The remaining two studies took slightly different approaches in their definition of delay intervals. Sadler and colleagues used patients’ referral routes to the hospital as surrogate measures of primary care delay [19]. They considered two-week waits (2WW) or ‘Direct Access Oesophago-gastro-duodenoscopy’ (OGD) as optimal routes to diagnosis, while waiting for routine appointments and acute admission were both considered sub-optimal. In addition, they reported ethnic differences in total diagnostic interval, which is the interval from first symptom to diagnosis. Similarly, Neal and Allgar reported the same interval, naming it pre-hospital delay [18]. For the rest of this paper, we have renamed these ‘the diagnostic interval’. They also reported ethnic differences in referral delay, which they defined as the waiting time from first GP visit to first hospital appointment.

#### Assessment of study quality

Overall, none of our seven studies had fewer than two concerns and none could be classified as “high quality” due to the systematic flaws in their methodology - as highlighted by the CASP assessment shown in Table 2. Only one study [18] had relatively low risk of bias, and was classified as “medium quality”. It had a large sample size, investigated several cancer sites, and classified ethnic groups in a way that was applicable to their target population. However, they only reported ethnic differences in pre-hospital delay, which includes factors relating to the patient, primary and secondary care and health care system making it difficult to ascertain where differences have arisen from. The remaining six studies had a greater overall risk of bias and were evaluated as “satisfactory”.

### Ethnic differences in delay intervals by cancer sites

#### Breast

Of the five studies that investigated ethnic differences in breast cancer diagnosis, two [16,18] found evidence of longer intervals in ethnic minority groups compared to the white majority. Neal and Allgar reported that ethnic minority women of Asian and black origin were more likely to experience longer diagnostic intervals, even after adjustment for marital status, gender, age and socioeconomic status (SES) (P < 0.001) [18]. Similarly, Velikova et al. found longer patient delay in Asian women compared to non-Asian (median of 61 days vs. median of 31 days, P = 0.005) even after adjustment for SES, age and health care settings [16]. Another study, Rajan et al., found that nearly half (45%) of the Asian women in their West Yorkshire (UK) cohort delayed over two months before presenting with breast symptoms [14]. They labelled this as ‘delay’ but without providing data for non-Asians. The remaining two studies [15,17] found no difference by ethnicity.

#### Oesophagogastric

Sadler and colleagues found evidence that Asians (13%) were less likely to take the optimal route to diagnosis (OGD and 2WW) compared to blacks (49%) and Caucasians (39%); P = 0.01 [19]. They also found that Asians (39%) and blacks (45%) were less likely to be diagnosed within three months of symptom discovery compared to Caucasians (63%); P = 0.03).

#### Colorectal

Neal and Allgar reported longer referral delay among Asians and Blacks even after adjustment for marital status, gender, age and SES (P = 0.02) [18]. No difference by ethnicity was found in pre-hospital delay.

#### Other cancer sites

For lung, prostate, NHL, and ovarian cancers no evidence was found of differences in delay interval across ethnic groups [18,20].

## Discussion

Health service provision must be culturally sensitive if health inequalities are to be reduced and eliminated. This is important in the UK (and other countries) as it becomes even more multicultural with considerable increases in ethnic minority population. Few UK studies have specifically explored ethnic inequalities in cancer diagnosis, and to the best of our knowledge no synthesis of evidence exists.

### Strengths and limitations

Our rigorous search strategy, and explicit inclusion/exclusion criteria, quality assessment of included studies and narrative synthesis followed best practice. Our search identified only a small number of studies, the bulk of which were (unsurprisingly) conducted in the UK, given that the UK is one of the most ethnically diverse countries in Europe. Our decision to omit American studies (other than Canada) was based on their very different health care system and ethnic groupings; it considerably reduced the number of selected studies, although this reflected our research question of whether there are ethnic inequalities in the time to diagnosis of cancer within a free health care system. A limitation was that most studies focused on breast cancer, reducing the scope of the review. Furthermore, any review is only as good as the studies it finds. Our selections differed considerably in methodology - especially in the definitions of intervals before diagnosis, which made quantitative synthesis inappropriate, and complicated the narrative synthesis. Participants’ ethnic groups were poorly defined in some studies; at times there was a mismatch between the ethnic groupings used for the study and contemporary ethnic groupings used in the whole population from which their sample was drawn [16,20]. We interpreted the CASP quality instrument for this review - primarily to fit our study topic: it is unlikely these minor changes led to more studies ‘failing’ our quality assessment. Finally, publication bias is possible as studies showing no association between ethnicity and cancer diagnostic delays may have failed to be published, leaving us with disproportionately positive studies.

### Comparison with previous studies

Two previous reviews [21,22] have examined delays in cancer diagnosis, although neither examined ethnicity. In both (like in the present review), the intervals were defined in a nonstandard way. This is understandable, as terminology in this subject was ill-defined before the advent of the Aarhus statement [13]. This provides more precise description of milestones along the cancer diagnostic pathway, as well as describing preferred research methodologies. In an assessment of primary care use prior to cancer diagnosis, Lyratzopoulos and colleagues found that ethnic minority groups were more likely to have consulted their GPs three or more times before hospital referral (odds ratio for Asian vs. white 1 · 73, 1 · 45 to 2 · 08; p < 0 · 0001; odds ratio for black vs. white 1 · 83, 1 · 51 to 2 · 23; p < 0 · 0001. This study did not report durations or intervals to diagnosis, so would not have met our inclusion criteria, though its results are consistent with our findings.

### Review findings

We found limited, methodologically-weak evidence for ethnic inequalities in cancer diagnosis, though largely for breast cancer. Conversely, the review found no evidence to suggest that ethnic minority groups were doing better at any stage of cancer diagnostic pathway. For breast cancer, three (of five studies) found longer patient and pre-hospital delays in ethnic minority women compared to their white counterparts; while the remaining two reported no difference. The latter two studies sampled participants from a relatively small number of women referred to a specialist clinic for further investigation. It is well-recognised that cancer patients may take alternative routes to diagnosis, such as routine specialist clinics or emergency admission; these will have been omitted from the two studies based in specialist clinics [23,24]. Furthermore, both studies were based on interviewing patients about their delay experiences whilst in the specialist waiting room. Therefore, their findings may have been influenced by selection and recall biases. In contrast, the three studies finding differences in diagnostic intervals across ethnic groups recruited participants with definite diagnosis of breast cancer, and then surveyed or examined their medical record for important dates of presentation and referral.

## Conclusion

Redressing inequalities in health (including those relating to ethnic diversity) is central to most reforms and strategies on health and social services in the UK. However, as the government progresses with the implementation of its austerity measures (with considerable cuts to health care expenditure) there are concerns that providers may prioritise differently. Potentially, some services including those intended to meet the specific health care needs of ethnic minority communities may be compromised - e.g., tailored services for language and communication needs, and the need for gender-specific providers. To ensure that policies are tailored to need, sufficient evidence is crucial. If existing evidence suggests ethnic variations in diagnostic delay, then policies aimed at promoting early diagnosis should also target the affected groups.

This review found some evidence for ethnic differences in cancer diagnosis with ethnic minorities taking longer to diagnosis. The evidence was largely in breast cancer patients. However, these findings are from methodologically heterogeneous studies, with different measures of delay. The studies had designs that are prone to bias. Future studies will need to examine the subject using a clearly defined and representative sample of ethnic minority groups, incorporate cancer types that are more common in ethnic minority groups and employ the definitions of cancer diagnostics in the Aarhus statement. This should be possible with the advent of large linked datasets containing routine healthcare data (e.g., the Diagnostic Imaging Dataset which incorporates ethnicity as a variable).

## Competing interests

The authors declare that they have no competing interests.

## Authors’ contributions

TM was involved in all aspects. WH participated in study design, data interpretation and preparation/revision of the manuscript. OU participated in study design, data extraction, assessment of study quality, and manuscript preparation. All authors read and approved the final manuscript.

## Pre-publication history

The pre-publication history for this paper can be accessed here:

http://www.biomedcentral.com/1471-2296/14/197/prepub
